# Molecular evidence of Tula virus in *Microtus obscurus* in the region of Yili, Xinjiang, China

**DOI:** 10.1186/s12879-019-4133-x

**Published:** 2019-06-14

**Authors:** Gang Guo, Baoping Guo, Xiran Wu, Yuanzhi Wang, Jianling Bao, Yuan Ren, Hongyu Li, Jun Li, Wenbao Zhang, Hua Yao

**Affiliations:** 1 State Key Laboratory of Pathogenesis, Prevention and Treatment of High Incidence Diseases in Central Asia, Clinical Medicine Institute, The First Affiliated Hospital of Xinjiang Medical University, Urumqi, 830054 Xinjiang China; 2 Xinjiang International Travel Healthcare Center, Urumqi, 830011 Xinjiang China; 3 College of Medicine, Shihezi University, Shihezi, 832003 China

**Keywords:** Tula virus, Hantavirus, Voles, *Microtus obscurus*, Genetic evolution, China

## Abstract

**Background:**

Hantaviruses are important zoonotic pathogens, and they pose a profound risk to public health. So far, there has been no evidence showing that Tula virus (TULV), one species of hantavirus, is endemic in China. In this study, we captured rodents and found that the Tula virus had infected voles in Yili region, Xinjiang, China.

**Methods:**

Rodents were captured by flooding their burrows in mountain pasture areas in Narati, Xinyuan County, Xinjiang, China. Hantavirus L gene fragments were amplified by nest RT-PCR using genus-specific primers. Positive samples were further identified by sequencing of RT-PCR products of S gene fragment for species identification. To identify the species of captured small mammals, the rodents’ cytochrome b (*Cyt*b) was amplified by PCR and sequenced. Phylogenetic analysis was used to show the clustering and evolution relationship of the viral nucleic acids.

**Results:**

Here, 31 out of 198 voles captured (16%) were infected with TULV. Host sequencing analysis showed these voles were *Microtus obscurus* (*M. obscurs*). Alignment and phylogenetic analysis of the exon region (1191 bp) of the hantavirus S gene confirmed that all of the detected amplicons were TULV, which was similar to one strain of TULV identified in Kazakhstan.

**Conclusion:**

This is the first identification of Tula virus in China, and we found that *M. obscurus* acts as a natural reservoir for carrying the virus. Although the infection rate in the local human population remains unknown, the high prevalence of TULV in the small mammals in the region constitutes a risk that this putative pathogen may spread to the local population.

**Electronic supplementary material:**

The online version of this article (10.1186/s12879-019-4133-x) contains supplementary material, which is available to authorized users.

## Background

Tula virus (TULV) is a Puumala-like virus, belongs to the genus hantavirus of family Bunyaviridae, and is primarily carried by *Microtus arvalis* [1]. TULV was initially discovered in *M. arvarlis* in Tula, Central Russia in the early 1990s [2]. Since then, *M. arvarlis* (*obscurus* [3]) has been found to act as a reservoir host of TULV in many European countries [4, 5], West Siberia [6], and Eastern Kazakhstan [7]. A few reports have shown human cases presenting a fever syndrome with severe headache [8] or hemorrhagic fever with renal syndrome and pulmonary involvement [9, 10]. However, most individuals with serological and/or pathogenic positive test were asymptomatic in European countries [11]. So far, no study has shown the epidemiological situation of TULV in China, even though voles are common rodents in some pasture areas in northern Xinjiang, China.

## Methods

### Sample collection and preparation

The investigation was performed in September 2015 in Narati mountain pastures in Xinyuan County, Yili region, Xinjiang. Voles were captured by flooding their burrows with a water pump. The voles that were captured alive were euthanized with barbiturate (100 mg/ kg). The voles were initially identified morphologically, then autopsied and approximately 1 g of lung tissue from each specimen was isolated and cut into 0.5-cm-thick slices using a pair of aseptic operation scissors and then placed to a sterile tube containing 5 mL of RNA stabilization reagent (Ambion RNAlater®, Life Technologies, Carlsbad, CA, US). Each tube was stored at 5 °C overnight, then transferred to − 80 °C for long-term storage. The capture of rodents in fields and protocols for using animals were approved by the Ethics Committee of the First Affiliated Hospital of Xinjiang Medical University, Urumqi, China (approval IACUC-2015).

### RNA extraction, cDNA synthesis, and PCR

Total RNA was extracted from the lung tissues of voles using TRIzol reagent (Thermo Fisher) according to the manufacturer’s instructions. The reverse transcription primer P14 (Table 1) was used for the first strand cDNA synthesis with the GoScript Reverse Transcription System (Promega, Beijing, China) [12, 16]. In brief, A PCR tube containing 2.0 μL of extracted RNA (500 ng/μL) and 1.0 μL of primer P14 (10 pmol/l) was heated in a 70 °C water bath for 5 min, then chilled in ice water for 5 min. After brief centrifugation, 2.0 μL of GoScript 5 × Reverse Transcriptase buffer, 0.5 μL of GoScript Reverse Transcriptase (200 U/μL), 0.5 μL of Recombinant RNasin Ribonuclease inhibitor (40 U/μL), 1 μL of PCR Nucleotide Mix (10 mM), 2.0 μL of MgCl_2_ (25 mM), and 1.0 μL of RNase-free deionized water were added to the tube. After incubation at 25 °C for 5 min, the tube was placed in a 42 °C water bath for 60 min. Reverse transcriptase was inactivated by heating to 70 °C for 15 min.

**Table 1:** List of PCR primers used within the study

Segment	Primer name	Primer sequence (5′-3′)	References
L,M,S	P14	TAGTAGTAGACTCC	[12]
L	Han-L-F1	ATGTAYGTBAGTGCWGATGC	[13]
	Han-L-R1	AACCADTCWGTYCCRTCATC	
	Han-L-F2	TGCWGATGCHACIAARTGGTC	
	Han-L-R2	GCRTCRTCWGARTGRTGDGCAA	
S	TULS1F	TAGTAGTAKRCTCCTTGAAAAGC	[14]
	TULS27F	TACTRAARCCGCTGGKATGA	
	TULS1760R	CGTGCATATATATAAGTGTACRGAGG	
Cytochrome B	CytB Uni fw	TCATCMTGATGAAAYTTYGG	[15]
	CytB Uni rev	ACTGGYTGDCC BCCRATTCA	

To identify hantavirus, the L gene segment (438 bp) was amplified by nest-PCR with viral-genus-specific primers, including outer PCR primers HAN-L-F1 and HAN-L-R1 and inner PCR primers HAN-L-F2 and HAN-L-R2 (Table 1) [13, 17]. All of the PCR products were analyzed using 2% agarose gel. To determine the species of the virus, S gene fragments were amplified by nest-PCR with cDNAs from L gene positive samples using reported primers in Table 1 [14].

### DNA extraction and molecular identification for species of hosts

Genomic DNA was extracted from approximately 30 mg of lungs of small mammals infected with hantavirus using a Tiangen TIANamp Genomic DNA Kit (Tiangen Biotech, Beijing, China) according to the manufacturer’s instructions. Complete cytochrome b (*Cyt*b) gene as a phylogenetic marker was amplified by PCR and the PCR products were subjected to sequencing [15].

### Phylogenetic analysis

The target nucleotide sequences were compared to sequences that were available in public databases using BLAST (http://blast.ncbi.nlm.nih.gov/Blast.cgi). The other hantavirus sequences for HTNV, DOBV, SEOV, SNV, ANDV, BAYV, PUUV, PHV, ISLAV, and LX307 (which should not be identified as TULV [18]) were set as outgroups. The phylogenetic tree was constructed using MEGA neighbor joining method (MEGA, version 7.0; http://www.megasoftware.net/) [19] and bootstrap values were 70%, calculated from 1000 replicates.

## Results

### Vole species identification

A total of 198 voles were captured in a Narati pasture (43°23′ N, 83°96′ E, altitude: above 1800 m) in Xinyuan County, Xinjiang, China. These small mammals came from 20 burrow-groups near the streams and each group contained 15–30 occupied burrows.

All of the voles were *M. obscurus,* initially classified by morphological identification*.* Two voles from hantavirus positive-groups that showed slight differences in morphology were further identified by PCR amplification and sequencing of *Cyt*b gene, which confirmed that the voles were *M. obscurus* with identical nucleotide sequences (GenBank accession No: KX058268-KX058269).

### Hantavirus detection and evolution analysis

**Fig. 1:**
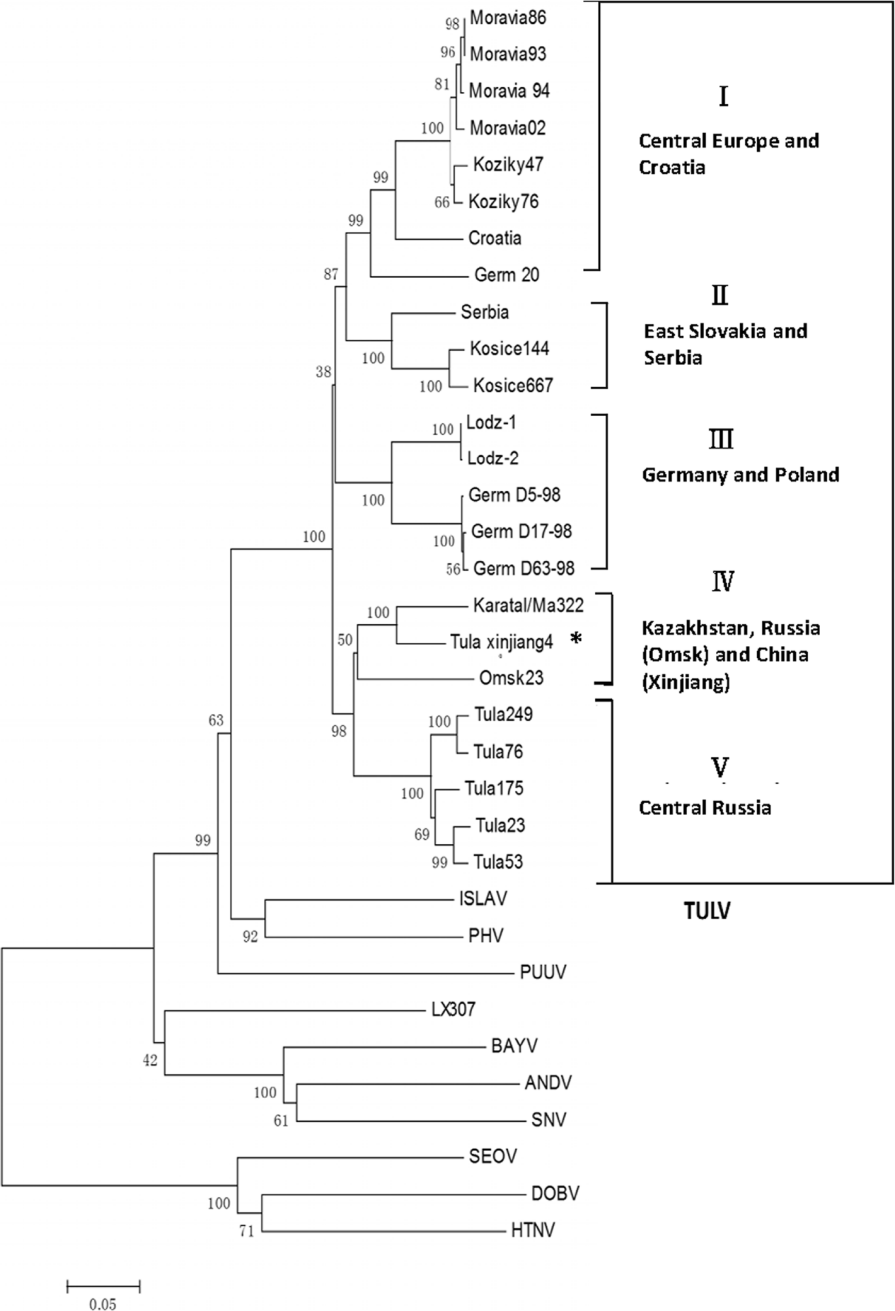
Phylogenetic tree of S gene fragment. The NJ tree was constructed with the S gene fragments (nt 43–1235). Tula virus, strain (GenBank accession number)/carrying host/Location: Tula249 (Z30944), Tula76 (Z30941), Tula175 (Z30943), Tula53 (Z30942), Tula23 (Z30945)*/*European common voles*/*Central Russia; Omsk23 (AF442621)/*Microtus gregalis*/West Siberia, Russia; Karatal322 (AM945877)/ *Microtus obscurus*/Kazakhstan; GermD5–98 (AF289819), GermD63–98 (AF289821), GermD17–98(AF289820)/*Microtus arvalis*/Germany; Lodz1 (AF063892), Lodz2 (AF063897)/*Microtus arvalis*/Poland; Kosice667 (Y13980), Kosice144 (Y13979)/European common vole/East Slovakia; Serbia (AF017659)/*Pitymys subterraneus*/Serbia-Yugoslaviaia; Germ20 (AF164093)/ *Microtus arvalis/*Germany; Croatia (AF164094)/*Microtus arvalis*/Croatia; Koziky47 (AJ223600), Koziky76 (AJ223601)/European common vole/Czech Republic; Moravia02 (Z49915), Moravia86 (Z48573), Moravia93 (Z48574); Moravia94 (Z48741)/*M. arvalis*/Czech Republic. Hantavirus, strain (GenBank accession number)/carrying host: HTNV, Hantaan virus, 76–118 (M14626)/*Apodemus agrarius*; DOBV, Dobrava-Belgrade virus, Dobrava (L41916)/*Apodemus flavicollis*; SEOV, Seoul virus, Z37 (AF187082)/*Rattus norvegicus*; SNV, Sin Nombre virus, Sin Nombre virus 77,734 (KF537003.1)/ *Peromyscus maniculatus*; ANDV, Andes virus, C9717869 (NC-003466)/*Oligryzomys longicaudatus*; BAYV, Bayou virus (L36929)/*Oryzonys palustris*; PUUV, Puumala virus (KT247597)/*Myodes glareolus*; PHV: Prospect Hill virus, (Z49098)/ *Microtus pennsylvanicus*; ISLAV, Isla Vista virus (U19302)/*Microtus pennsylvanicus*; LX307, unconfirmed hantavirus (HM756286)/*Eothenomys milemiletus*/Yunnan Province, China. *virus strain reported in this article

Hantavirus L gene fragments were successfully amplified and sequenced from 31 out of 198 voles, such that 16% of voles were infected with hantavirus. We further amplified and sequenced the S gene of hantavirus from the two L-gene-positive samples to determine the species of the viruses. The sequence analysis showed the nucleotide sequences of two isolates are identical, called Tula xinjiang4 (GenBank accession No: KX270414). Combining with the sequences published in the GenBank, the phylogenetic analysis showed 5 genetic lineages (I–V) likely based on the geographic distribution in Eurasia (Fig. 1). Tula xinjiang4 is very similar to Karata322 (isolated from eastern Kazakhstan, altitude: 1200 m) [7] with which it has been found to share 92.1% nucleotide identity and 98.2% amino acid identity, and Omsk23 (isolated from southwest region of Siberia in Russia), with which it has been found to share 87.5% nucleotide identity and 97.5% amino acid identity. The referenced sequences were isolated from European common voles, *M. arvalis* (*obscurus*), *M. gregalis,* and *Pitymys subterraneus*, respectively. (Additional file 1: Table S1 and Table S2).

## Discussion

Few studies have explored the epidemiology of hantaviruses in Xinjiang. Only Seoul virus (SEOV, a hantavirus), found in *Rattus norvegicus* in Urumqi, Xinjiang, has been assessed in this way [20, 21]. The present study showed for the first time that *M. obscurus* [3, 22] carries TULV in China. The distribution of hantavirus is likely related to the distribution of rodents [1, 23]. In Xinjiang, there are 69 species of rodents, which belong to 10 families and 34 genera, accounting for 40% of rodent species in China [23, 24]. Among the small mammals, there are 19 species in subfamily *Microtinae* and 7 species in the genera *Microtus* [23]. In this way, it is likely that other voles or small mammal species may also serve as hosts for TULV or hantavirus in Xinjiang. This needs further investigation.

Hantavirus infection is a zoonosis and it remains a severe public health problem. However, very few patients infected with TULV in Europe have shown symptoms [8–10] with the most serologically positive cases being asymptomatic [11]. It may be relevant whether the populations in these areas where *M. obscurus* are likely to be infected with TULV.

The two locations, Yili in China and eastern Kazakhstan, where the TULV-infected voles were found, are geographically close and have similar landscapes; both are in the Tianshan Mountains. Viral sequence analysis showed TULV Xinjiang4 to be very similar to Karata322 and the overall lineages appeared closely related to those isolates from central Russia and Siberia, indicating these viruses share a common ancestor (bootstrap support value 98%, on the NJ-tree).

**Fig. 2:**
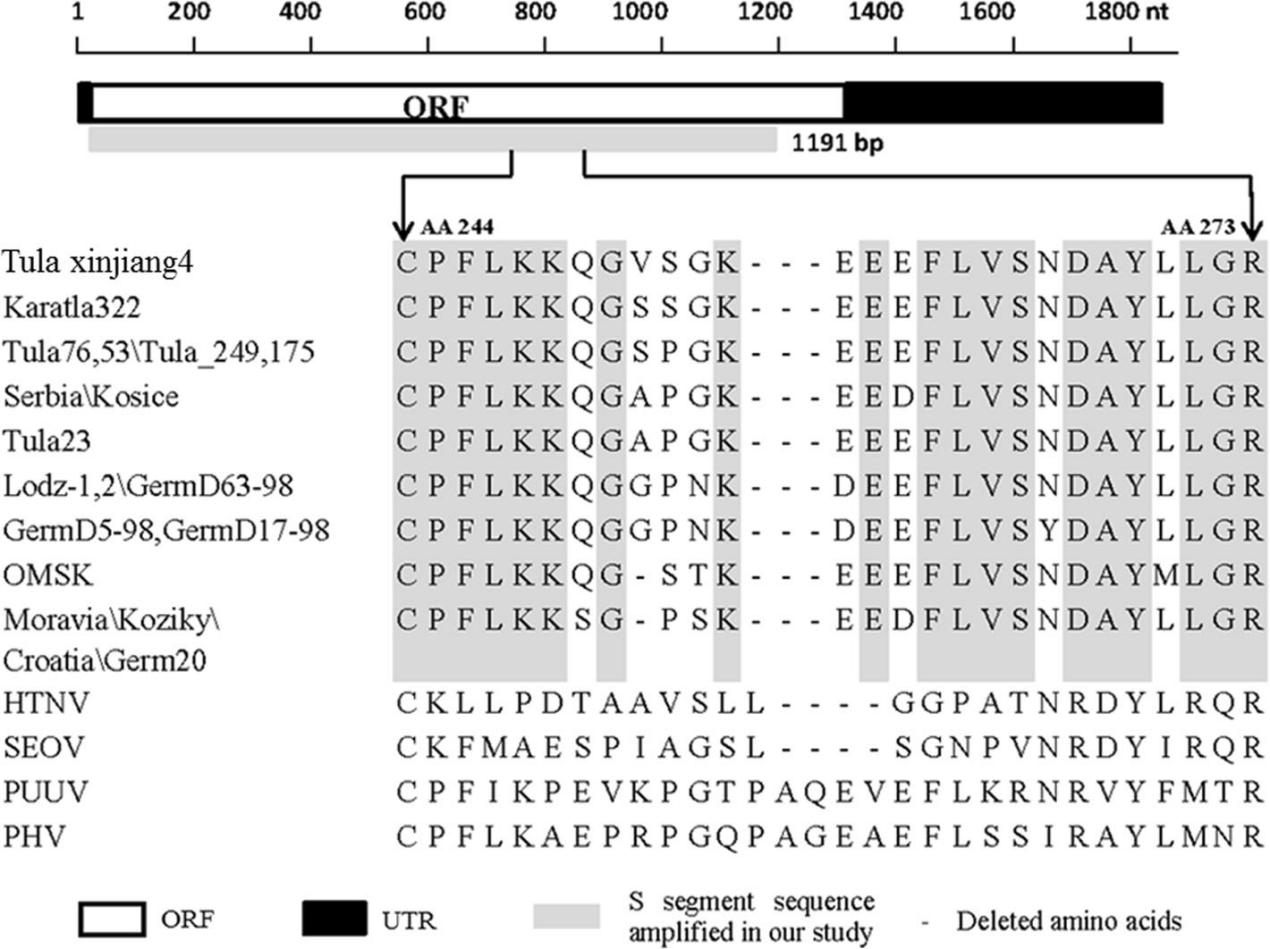
Alignment of partial nucleocapsid protein sequences of Tula virus (TULV). Amino acids that matched those of TULV are highlighted in gray. ORF: Open Reading Frame; UTR: Untranslated Region

Hantavirus contains three genes, negative-stranded large (L), middle (M), and small (S) genomic RNA segments encode a polymerase, M proteins composed of two surface glycoproteins (Gc and Gn), and the nucleocapsid protein (N protein), respectively. The complete S segment sequence of TULV strain is 1830 nt in size and the sequence includes the following structures: a 5′-untranslated region (UTR, nt 1–42), an open reading frame for the 430 amino acids (AA-long N protein, nt 43–1335) and a 3′-UTR (nt 1336–1830) [7]. In our study, the predicted N-protein sequence revealed that the N protein sequence region (amino acid, AA 244–273) of TULV contains a highly variable region. There are 3 AA (AA 256–258) deletions in Tula Xinjiang4 and other TULV strains, 4 AAs deleted in hantavirus (HTNV) and SEOV (AA 257–260) compared with Prospect Hill virus (PHV) and Puumala virus (PUUV) (Fig. 2). It may be useful to determine whether the deletion of these sequence impacts the function of N protein.

## Conclusions

TULV was identified in Narati pasture, Yili region, Xinjiang, western China by molecular detection. The high prevalence of the virus in *M. obscurus* indicates Tula virus is naturally transmitted in this area. It may be useful to determine whether this virus infects other hosts, such as humans.

## Additional file

Additional file 1: **Table S1**. Identities and differences among nucleotide sequences of TULV virus strains from Eurasia. **Table S2**. Identities and difference among amino acid sequences deduced for TULV virus strains from Eurasia. (DOC 1366 kb)

## Data Availability

All of the data generated or analysed during this study are included in this published article. The sequences generated have been submitted to GenBank under accession numbers KX058268 to KX058269 for *Cyt*b gene sequences and KX270414 for the partial segment of S genomic exon sequence.

## Abbreviations

AA: Amino Acid
*Cyt*b: Cytochrome b
MEGA: Molecular Evolutionary Genetic Analysis
ORF: Open Reading Frame
RT-PCR: Reverse Transcription-Polymerase Chain Reaction
TULV: Tula Virus
UTR: Untranslated Region
