# Supplementary material for: Molecular evidence of Tula virus in Microtus obscurus in the region of Yili, Xinjiang, China
Source: BMC Infect Dis. 2019 Jun 14;19:527. doi: 10.1186/s12879-019-4133-x (PMC6570900; doi:10.1186/s12879-019-4133-x)
Supplement: Supplementary file 1 — Table S1. Identities and differences among nucleotide sequences of TULV virus strains from Eurasia. Table S2. Identities and difference among amino acid sequences deduced for TULV virus strains from Eurasia. (DOC 1366 kb) [file 12879_2019_4133_MOESM1_ESM.doc]

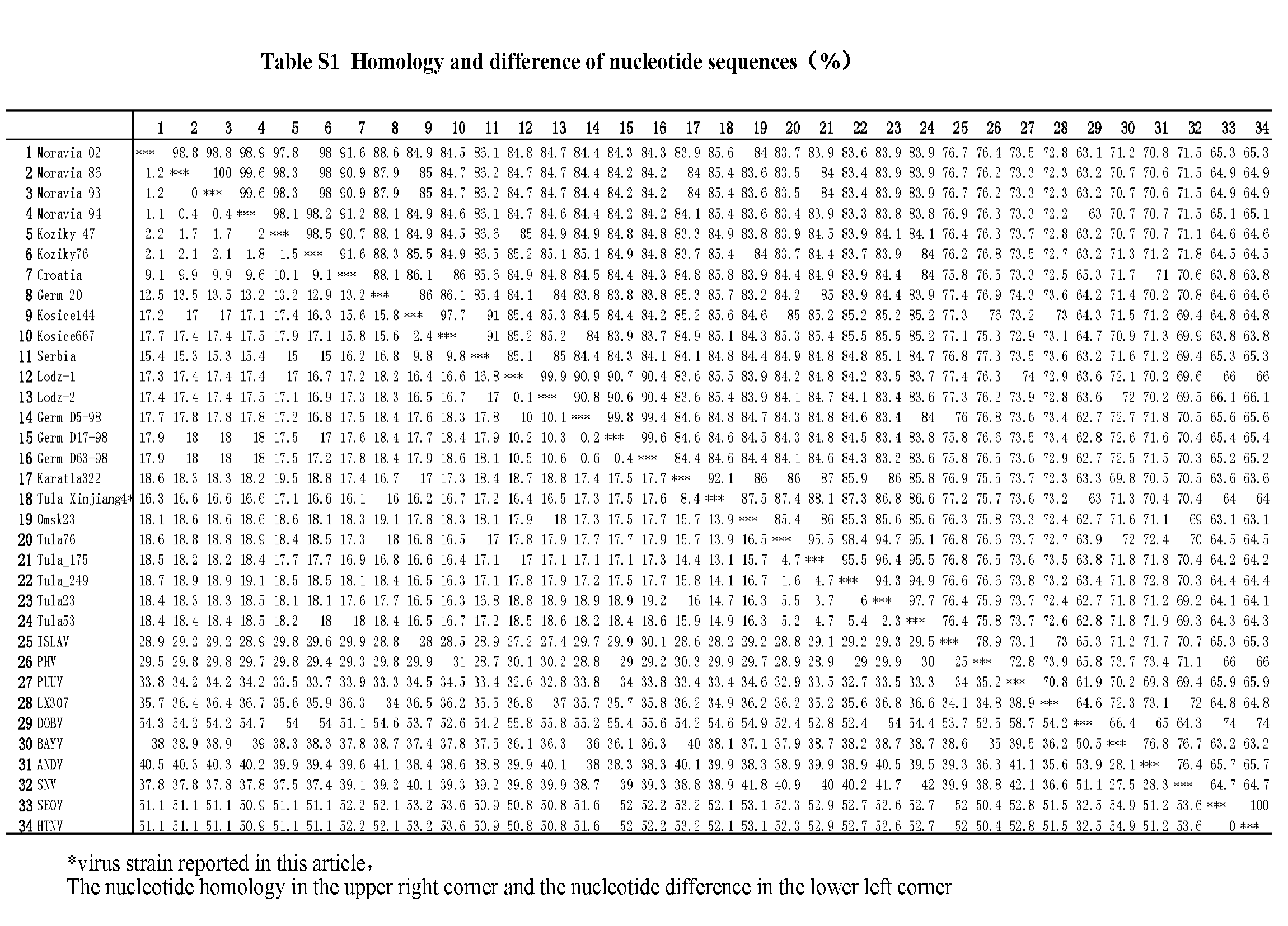


*Virus strain reported in this article

The nucleotied homology in the upper right corner and amino acid difference in the lower left corner


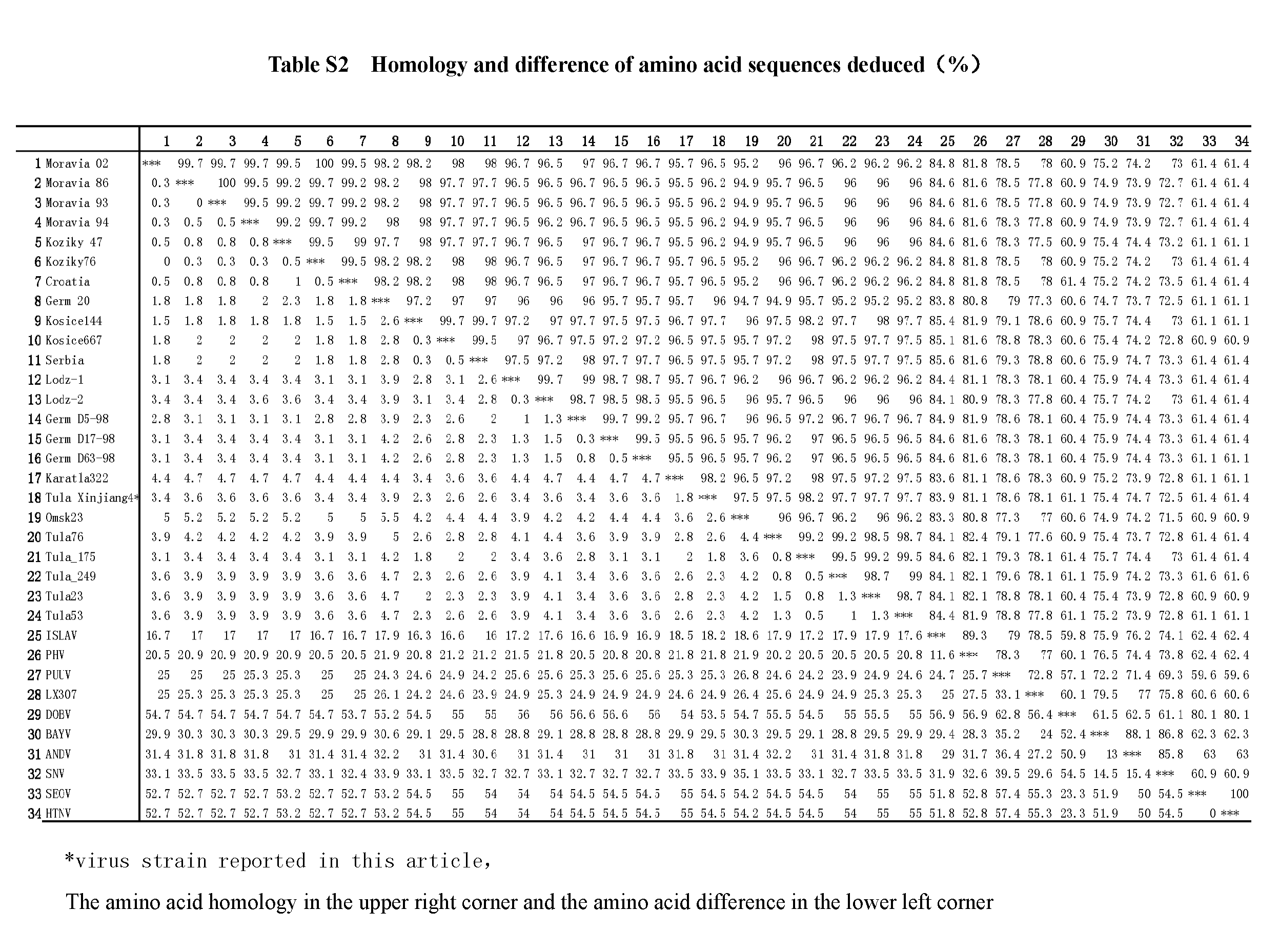


*Virus strain reported in this article

The amino acid homology in the upper right corner and amino acid difference in the lower left corner
